# Inside‐Out IP_3_‐Mediated G Protein‐Coupled Receptor Activation Drives Intercellular Ca^2+^ Signaling in the Vascular Endothelium

**DOI:** 10.1096/fj.202500370RR

**Published:** 2025-07-10

**Authors:** C. Buckley, X. Zhang, M. D. Lee, C. Wilson, J. G. McCarron

**Affiliations:** ^1^ Strathclyde Institute of Pharmacy and Biomedical Sciences University of Strathclyde Glasgow UK

**Keywords:** blood vessels, calcium signaling, calcium waves, cell communication, endothelium, GPCR, vascular tone

## Abstract

The endothelium's control of nearly all vascular function relies on rapid intercellular communication to coordinate cellular activity across scale. A key form of intercellular communication arises from the regenerative propagation of IP_3_‐evoked Ca^2+^ signals from cell to cell, which regulate vessel tone, modulate vascular permeability, and determine immune responses. Despite their importance, the mechanisms by which regenerative propagation of IP_3_‐evoked Ca^2+^ signals occurs are poorly understood. Here, in intact resistance arteries, precision photolysis of IP_3_ combined with high‐resolution mesoscale imaging, targeted drug application, and advanced analytical techniques was used to determine the mechanisms underlying regenerative propagation of IP_3_‐evoked Ca^2+^ signals in the endothelium. Elevated IP_3_ in the initiating cell triggers a noncanonical inside‐out signaling mechanism that leads to transcellular activation of a G_αq/11_‐coupled receptor in a neighboring (receiving) cell. This, in turn, initiates canonical outside‐in signaling via PLC, leading to the hydrolysis of PIP_2_ and production of IP_3_. This process creates a regenerative, IP_3_‐dependent signaling cascade operating between adjacent cells. Notably, neither Ca^2+^ nor IP_3_ diffusion through gap junctions plays a significant role in intercellular communication. Our findings uncover a previously unrecognized mechanism of endothelial communication, in which noncanonical IP_3_‐driven transcellular activation of G protein‐coupled receptors sustains a regenerative signaling loop, highlighting a novel framework for intercellular coordination in the vascular endothelium.

## Graphical abstract

During endothelial cell Ca^2+^ wave propagation, IP_3_ in the initiating cell activates IP_3_Rs on the endoplasmic reticulum, releasing Ca^2+^ into the cytoplasm. We show that IP_3_ is required for signal propagation to neighboring cells and propose that this is due to activation of membrane‐bound pore‐dead IP_3_Rs, as part of a noncanonical signaling inside‐out signaling pathway. We further show that this activates a GPCR on the receiving cell membrane, releasing the G_αq/11_ subunit to bind to PLC and mediate the breakdown of PIP_2_ into IP_3_, in an outside‐in signaling process. IP_3_ then binds the IP_3_R on the ER in the receiving cells, releasing Ca^2^^+^.

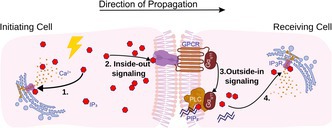

## 1 Introduction

The endothelium, a critical regulator of vascular function, relies on precise intercellular communication to manage signals that control blood vessel diameter, permeability, angiogenesis, and immune responses. A fundamental mechanism underlying this communication is the regenerative propagation of IP_3_‐evoked Ca^2+^ signals among cells to enable coordination of endothelial activity. However, the mechanisms triggering regenerative IP_3_ production and subsequent signal propagation are poorly understood, limiting our ability to address vascular dysfunctions associated with diseases like hypertension and neurodegenerative conditions. We describe a novel mechanism for intercellular communication, whereby noncanonical IP_3_ activation of a transcellular G protein‐coupled receptor (GPCR) drives regenerative IP_3_ production in neighboring cells.

Cardiovascular function is monitored and adjusted by the endothelium in response to a multitude of incoming signals that act on endothelial cells. The endothelium manages the signaling load by using cells that are specialized to detect specific stimuli, which then communicate with neighboring endothelial cells to transmit information and coordinate changes in endothelial function across scale [1]. This “transcellular” information transfer between endothelial cells is encoded in concentration levels of second messengers such as IP_3_ and Ca^2+^.

An important mechanism driving increases in intracellular Ca^2+^ is the opening of IP_3_ receptors (IP_3_Rs) on internal stores, principally the endoplasmic reticulum. This canonical signaling cascade is typically initiated by ligand binding to GPCRs on the outer membrane of cells, inducing a conformational change in the G protein which allows the subunit to dissociate and initiate downstream signaling pathways. There are four prominent classes of G protein families, with Gα_q/11_ being the primary subfamily involved in endothelial IP_3_‐mediated Ca^2+^ release [2, 3]. Blood vessel activators that act on the endothelium, for example, acetylcholine (ACh), bind Gα_q/11_ GPCRs, such as the M3 muscarinic receptor, stimulating phospholipase Cβ (PLCβ) to hydrolyze membrane‐bound phosphatidylinositol‐4,5‐biphosphate (PIP_2_). PIP_2_ splits into diacylglycerol (DAG) and inositol‐1,4,5‐triphosphate (IP_3_). The free IP_3_ binds and opens IP_3_Rs on the endoplasmic reticulum, allowing Ca^2+^ release from the internal stores.

Alterations in Ca^2+^ concentrations within single endothelial cells are translated into endothelial‐wide coordinated responses by the propagation of Ca^2+^ gradients (“Ca^2+^ waves”) between cells [4]. The mechanisms which act to scale local intracellular signals to drive extended cell–cell communication remain surprisingly obscure and contentious. Notwithstanding, traditional hypotheses propose that signaling occurs in one of two ways; paracrine diffusion of molecules like ATP, or by the transcellular movement of IP_3_ through gap junctions. Paracrine diffusion entails the release and diffusion of a factor into the extracellular space to bind to a receptor on the plasma membrane of a neighboring cell, triggering downstream intracellular signaling [5]. In the case of diffusion through gap junctions, IP_3_ [6, 7, 8] flux will be driven between cells by the electrochemical driving force acting on the inositide to trigger Ca^2+^ release from the internal stores in neighboring cells [9]. Since IP_3_ is a trivalent anion, in addition to changes in the concentration gradient of the inositide, changes in transmembrane potential should exert influence in the transcellular flux of IP_3_ between coupled cells. However, both models (paracrine signaling and gap junction coupling) fail to fully explain the dynamics observed in the vascular endothelium. For example, paracrine communication is unlikely to transmit significant signaling against the direction of blood flow.

Recently, we proposed that a novel mechanism accounts for cell communication [10]. In the vascular endothelium, IP_3_ is sufficient to evoke a regenerative Ca^2+^ wave propagation across cells in direct physical contact, without relying on diffusion of either IP_3_ or Ca^2+^ via gap junctions. Instead, IP_3_ appears to activate an unidentified PLC “bridge” that mediates signal transmission between neighboring adjacent cells. The bridge either directly or indirectly activates PLC, leading to the breakdown of PIP_2_ and the generation of IP_3_. Consequently, IP_3_ mediates IP_3_ production in adjacent cells to regeneratively propagate the Ca^2+^ signal. The question arises as to how IP_3_ generates IP_3_ production, and the role played by IP_3_ receptors. Since agonist‐induced IP_3_ production is initiated by GPCR activation, we hypothesized that IP_3_ may activate a GPCR to facilitate IP_3_ generation during the propagation of endothelial Ca^2+^ waves.

Here, using a cutting‐edge imaging approach that allows precise activation or deactivation of specific subgroups of cells in intact resistance arteries, we demonstrate that an increase in IP_3_ in one cell activates Gα_q/11_‐coupled GPCRs in neighboring cells. This triggers PLC‐mediated hydrolysis of PIP_2_ into IP_3_ in neighboring cells, enabling regenerative, IP_3_‐driven signal transmission between endothelial cells. Importantly, this propagation does not depend on gap junctions or electrochemical gradients.

These findings reveal a previously unrecognized pathway for intercellular communication and provide critical insights into the complexity of mechanisms driving vascular signaling.

## 2 Materials and Methods

### 2.1 Animals

All animal husbandry and sacrifice were carried out in accordance with the prior approval of the University of Strathclyde Animal Welfare and Ethical Review Body and under relevant UK Home Office Regulations (Schedule 1 of the Animals [Scientific Procedures] Act 1986, UK). Strathclyde biological procedures unit is a conventional facility that undertakes FELASA quarterly health monitoring. Animal studies are reported in compliance with the ARRIVE guideline [11].

Male Sprague–Dawley rats (10–12 weeks old; 250–300 g), from an in‐house colony, were used for the study. The animals were housed 3 per cage and the cage type was North Kent Plastic model RC2F with nesting material “Sizzle Nest.” A 12:12 light dark cycle was used with a temperature range of 19°C–23°C (set point 21°C) and humidity levels between 45% and 65%. Animals had free access to fresh water and SDS diet RM1 (rodent maintenance). The enrichment in the cages was aspen wood chew sticks and hanging huts. Animals were euthanized by cervical dislocation and death confirmed by exsanguination. The mesenteric bed was removed. All experiments were performed using first‐ to third‐order mesenteric arteries. Controls and experimental treatments were carried out in the same tissue, so blinding and randomization were not used. Group sizes were designed to be equal.

### 2.2 Mesenteric Artery Preparation and Mounting

To prepare *en face* blood vessel preparations, dissected arteries were cleaned of fat and connective tissue and cut open longitudinally to expose the endothelial layer. Arteries were then pinned flat in custom‐designed baths, with a Sylgard base, using 50 μm diameter pins. Dissection and experiments were carried out in a physiological saline solution (PSS: 145 mM NaCl, 2 mM MOPS, 4.7 mM KCl, 1.2 mM NaH2PO4, 5 mM Glucose, 0.02 mM EDTA, 1.17 mM MgCl, 1 mM CaCl, pH 7.4). In some experiments, Ca^2+^ free PSS (145 mM NaCl, 2 mM MOPS, 4.7 mM KCl, 1.2 mM NaH2PO4, 5 mM Glucose, 0.02 mM EDTA, 2.34 mM MgCl,1 mM EGTA, pH 7.4) and high K^+^, Ca^2+^ free‐PSS (2 mM MOPS, 134.4 mM KCl, 1.2 mM NaH2PO4, 5 mM Glucose, 0.02 mM EDTA, 1.17 mM MgCl, pH 7.4) were used.

Endothelial cells were loaded with the Ca^2+^ indicator dye Cal520/AM (5 μM in PSS with DMSO and 0.02% Pluronic F‐127) for 30 min at 37°C and then mounted in a custom‐designed flow chamber [12].

### 2.3 Endothelial Patch Isolation

Mesenteric arteries (first to third order) were dissected, the fat removed, and cut open longitudinally. Any blood that remained on the endothelial surface was carefully removed. Arteries were cut into five strips of approximately 2 mm length, suspended in PSS with collagenase (Type 2, 256 units/mg, 2 mg.mL^−1^), and enzymatically digested in a water bath at 37°C for 45 min. The supernatant was removed, and a wide‐bored, fire‐polished glass pipette was used to gently triturate arteries and isolate the endothelial cell patches. Patches were transferred to an 8‐well chamber slide (μ‐slides; Ibidi, Germany) for 3 h before fixation for immunocytochemistry.

### 2.4 Image Acquisition

#### 2.4.1 Imaging System 1

A Nikon Eclipse FNI upright microscope equipped with a Nikon Fluor 16X 0.8 NA water immersion objective lens and a pE‐4000 CoolLED system (365/490/550/635 nm excitation) and a quad DAPI/FITC/TRITC/Far Red filter set. Images were acquired using a Photometrics Evolve 13 EMCCD camera (1024 × 1024). All images were acquired using MicroManager v2 [13] for 2 min at 10 Hz.

The microscope rig was equipped with a hydrostatic pressure ejection system (Pneumatic PicoPump PV820, World Precision Instruments, Sarasota, FL, USA), allowing focal application of drugs from a puffer pipette that was positioned ~50 μm above the surface of the blood vessel, perpendicular to the direction of the flow. See “Localized Pressure Ejection Experiments” section.

#### 2.4.2 Imaging System 2

A Nikon Eclipse TE300 inverted microscope fitted with a CoolLED pE‐300 LED illumination system (400/490/550 nm excitation) and custom designed DAPI/FITC/TRITC filter set. A 40X 1.3 NA Nikon S Fluor oil‐immersion objective lens was used for Ca^2+^ imaging experiments, and a 100X 1.3NA Nikon S‐Fluor lens for imaging immunocytochemistry on endothelial patches. Images were acquired using an Andor iXon EMCCD camera (1024 × 1024) and MicroManager v1.44 [14].

### 2.5 Acetylcholine Application

In experiments where acetylcholine (ACh) was used to elicit Ca^2+^ responses, directional flow (1.5 mL.min^−1^) was used throughout the experiment. The directional flow consisted of either PSS (control) or ACh (100 nM) in PSS. After ACh application, arteries were washed (with PSS) for 10 min to ensure ACh removal, followed by a 5 min rest period before subsequent recordings.

To visualize Ca^2+^ activity, images were created by calculating Δ*F*/*F*_0_ for each image in the recording. A maximum intensity projection of 10 s (100 frames) following ACh application was taken and presented using a time‐correlated colormap.

### 2.6 Localized Uncaging

In experiments in which endothelial Ca^2+^ responses were evoked by photolysis of caged IP_3_, the endothelium was loaded with Cal520/AM (5 μM in PSS with DMSO and 0.02% Pluronic F‐127) and a membrane‐permeant Ins(1,4,5) P3‐caged IP_3_ (5 μM; cIP_3_) for 30 min at 37°C [15, 16].

Laser photolysis of cIP_3_ was achieved using a Rapp OptoElectronic DL‐Series UV (375 nm) laser coupled into a Firefly system, with a UGA‐42 scanner, to set the uncaging region via the software. This system was mounted onto Imaging System 1. The UV photolysis light was first passed through an attenuating neutral density filter (1% transmission) and used at a power such that it was 2 mW before loss as it traveled through the optics. The region of interest was determined using the acquisition frames over which the laser had been used. In this experimental setup, experiments blocking PIP_2_ generation used LY294002 (300 μM) and wortmannin (50 μM), and GPCR function was inhibited using YM254890 (1 μM), or FR900359 (5 μM), or NF 449 (1 μM), or Gallein (5 μM), or Pretussis toxin (PTX 100 ng.ml^−1^).

In other experiments, flash photolysis of cIP_3_ was achieved using a Rapp Optoelectronics flash lamp (00‐325‐JML‐C2) at 200 V, which produced light of ~1 ms duration. This system was mounted onto Imaging System 2. The flashlamp output was passed through a 395 nm short pass filter into a 1250 μm diameter light guide. The light guide was coupled to the epi‐illuminator of the TE300 microscope, and the output focused on the endothelium using broadband light. For each imaging session, broadband light was used to identify the photolysis region (~70 μm diameter). In this setup, experiments blocking gap junction function using Gap 27 (300 μM) were performed.

Endothelial Ca^2+^ activity induced by cIP_3_ photolysis was imaged at a rate of 10 Hz. Baseline Ca^2+^ activity was recorded for 30 s prior to the photolysis of cIP_3_. To ensure proper Ca^2+^ store refilling, all cIP_3_ uncaging experiments were performed with a minimum of 15 min rest between each photolysis event.

To visualize Ca^2+^ wave propagation, images of active Ca^2+^ wavefronts were created by calculating ΔF/F_0_ for each image in the recording. For cIP_3_‐evoked Ca^2+^ experiments, a maximum intensity projection of the first 5 s (50 frames) immediately following uncaging was taken and presented using either a green or a time‐correlated colormap. Propagation area was calculated by applying a Gaussian blur (*σ* = 10) to the maximum intensity projections, thresholding the image, and creating a mask from which the area was measured. Since experiments were paired (unless otherwise stated), images were contrast‐matched for control and treatment.

### 2.7 Localized Pressure Ejection Experiments

In some experiments, BAPTA/AM (30 μM), U73122 (10 μM) or high K^+^ (134 mM, Ca^2+^ free) PSS were focally applied (or “puffed” on) to the blood vessels via pressure ejection from a puffer pipette. In these experiments, a control cIP_3_‐initiated Ca^2+^ response was first recorded, and the vessels were allowed to reequilibrate for 15 min. For BAPTA/AM and U73122 this was performed in PSS; for high K^+^ (Ca^2+^ free) this was performed in Ca^2+^ free PSS to prevent smooth muscle contraction.

The puffer pipette solution contained a pharmacological agent (BAPTA/AM (30 μM) or U73122 (10 μM) or high K^+^ (134 mM, Ca^2+^ free) PSS) and a fluorophore (sulforhodamine B, 1 μM) to visualize the region‐of‐influence of the puffer‐ejected solution. After the puffer pipette was positioned ~50 μm from the endothelial surface, directional flow (1.5 mL.min^−1^) of bath solution was initiated and maintained throughout the experiment to limit the spread of the drugs. In BAPTA/AM and U73122 experiments, the bath solution was PSS; in high K^+^ PSS experiments, the bath solution was Ca^2+^‐free PSS.

Pharmacological agents were puffed onto the blood vessels via pressure ejection for a period of time appropriate to the experiment; for BAPTA/AM and U73122 experiments this was 15 min, for high K^+^ PSS experiments this was long enough to stabilize flow, ~2 min. Simultaneously, cIP_3_‐evoked Ca^2+^ activity was imaged in the FITC channel (490 nm excitation) and the sulforhodamine B signal in the TRITC channel (550 nm excitation) at 5 Hz per channel. During the rest period between each experiment, arteries were washed with PSS to replenish internal Ca^2+^ stores.

Puffer pipette locations, bath PSS flow direction, photolysis site, and puffing region are shown and labeled in each image presented. To visualize Ca^2+^ wave propagation, images of active Ca^2+^ wavefronts were created by calculating ΔF/F_0_ for each image in the recording. For cIP_3_‐evoked Ca^2+^ experiments, a maximum intensity projection of the first 5 s (50 frames) immediately following uncaging was taken and presented using either a green or a time‐correlated colormap. Propagation area was calculated by applying a Gaussian blur (*σ* = 10) to the maximum intensity projections, thresholding the image, and creating a mask from which the area was measured. Since experiments were paired (unless otherwise stated), images were contrast matched for control and treatment.

### 2.8 Ca^2+^ Signal Analysis

ACh‐ or cIP_3_‐evoked Ca^2+^ signals were measured in each cell as previously described [12]. In brief, automated Fiji macros were used to extract cell coordinates and track cell positions between datasets. Single‐cell Ca^2+^ signals were then measured from each cell and processed using a custom algorithm written in the Python programming language [12, 17, 18]. Raw fluorescence (*F*) signals were converted to baseline‐corrected fluorescence intensity (*F/F*_0_) by dividing each intensity measurement by the average value of a 100‐frame baseline period at the start of each trace. *F/F*_0_ signals were smoothed using a 21‐point third order polynomial Savitzky–Golay filter, and key signal parameters (e.g., amplitude, frequency, number of cells, time of event) were extracted automatically. Analyses of cIP_3_‐evoked Ca^2+^ responses were performed either within the photolysis region, within the perfusion region, or for the entire field of view (FoV).

To visualize and measure propagation speed, cell coordinates and the time of the first recorded Ca^2+^ peak were extracted. These values were imported into MATLAB R2023b using custom‐written code, and the distance versus time plot was generated. A colormap was applied to the time of first activation to produce a color‐coded map of coordinates and activation times. The effective speed of signal propagation was calculated by measuring the distance from the uncaging region and the time of first activation.

Analysis of signals in the photolysis region was achieved by applying a mask to the data. The photolysis region was measured from an image acquired during the activation of the flash lamp or laser. For measurements in the region where drugs were applied via pressure ejection, a mask was created using the sulforhodamine signal. Parameters such as the number of active cells within the RoIs, Ca^2+^ response, and propagation area were then extracted.

### 2.9 FluoVolt Membrane Potential Experiment

The endothelium was loaded with FluoVolt (1X dye, 10X PowerLoad concentrate, 8 min room temperature) and mounted on imaging system 1.

A bath solution of Ca^2+^‐free PSS was flowed onto the vessel and a control cIP_3_ photolysis recording was acquired. The bath solution was changed to PSS for 15 min to refill the Ca^2+^ stores, then the bath solution was changed to Ca^2+^ free PSS. A perfusion pipette solution of High K^+^ (134 mM, Ca^2+^ free) PSS with sulforhodamine B (1 μM) was puffed over the photolysis region (as described above) as a second cIP_3_ photolysis recording was taken. The bath solution was changed to PSS for 15 min to refill the Ca^2+^ stores, then the bath solution was changed to high K^+^ (134 mM, Ca^2+^ free) PSS, and a final cIP_3_ photolysis recording was taken.

Images were acquired at 5 Hz per channel, recording the FluoVolt (490 nm excitation) and sulforhodamine (550 nm excitation) channels simultaneously. To acquire traces of the change in fluorescence, regions of interest (indicated in the images shown) were drawn and signals extracted, smoothed using a rolling average of 20, and normalized to the baseline signal.

### 2.10 Fluorescent Recovery After Photobleaching (FRAP) Experiments

The endothelium was loaded with calcein/AM (0.02% Pluronic F‐127 in PSS) for 30 min at 37°C. FRAP experiments were performed on imaging system 1 using a ROE DL‐Series UV (375 nm) with a UGA‐42 scanner (Rapp OptoElectronics). The FRAP region was selected via the controlling software. A 40X 0.8NA water‐immersion lens was used in these experiments, and the power was measured at approximately 8 mW. The scan was repeated ~5–10 times in ~5 cells to reduce the fluorescence intensity within the FRAP region to ~70% of the initial value. Images were then acquired at 0.1 Hz for 1 h to image the recovery period.

### 2.11 Immunocytochemistry

Freshly isolated endothelial cell patches or *en face* arteries were fixed in 4% paraformaldehyde (PFA; Agar Scientific, UK) in phosphate buffered saline (PBS) (20 min, room temperature), refreshing the PFA after 10 min. Cells were washed (5 min) three times in glycine solution (0.1 M), three times in PBS (5 min), permeabilized with Triton‐X100 (0.2% in PBS; 30–45 min), washed three times in PBS (5 min), three times in an antibody wash solution (0.15 M NaCl, 15 mM Na3C6H5O7, 0.05% Triton‐X100 in milliQ water; 5 min), and incubated with blocking solution (5% donkey serum in antibody wash solution; 1 h at room temperature). Cells were then incubated overnight at 4°C with an anti‐CD31 (PECAM) primary antibody (R&D Systems cat. # AF3628, RRID:AB_2161028, 1:1000, raised in goat), anti‐α tubulin (Sigma, Cat # T5168, RRID: AB_477579, 1:1000, raised in mouse) and anti‐IP_3_R (Millipore, Cat. # 07–1210, RRID:AB_1587207, 1:100, raised in rabbit) primary antibodies, each diluted in antibody buffer at the concentrations stated (0.15 M NaCl, 15 mM Na3C6H5O7, 2% donkey serum, 1% BSA, 0.05% Triton X‐100, 0.02% sodium azide in milliQ water). All antibodies were used once. Vessels were washed three times in antibody wash solution (5 min), and incubated with fluorescent secondary antibodies conjugated to the appropriate combination of Alexa Fluor 488 (donkey anti‐goat, Cat. # A‐11055, RRID:AB_2534102), Alexa Fluor 568 (donkey anti‐goat, Cat. # A‐11057, RRID: AB_2534104), Alexa Fluor 555 (donkey anti‐rabbit, Cat. # A‐31572, RRID: AB_162543), Alexa Fluor 647 (donkey anti‐rabbit, Cat. # A‐31573, RRID: AB_2536183), and Alexa Fluor 488 (donkey anti‐mouse, Cat. # A‐ 21202, RRID: AB_141607) all Invitrogen, 1:1000 dilution, in antibody buffer (1 h at room temperature). Cells were washed three times in antibody wash solution, incubated with the nuclear stain, 4′,6‐diamidino‐2‐phenylindole (DAPI; 4 nM; 5 min), and washed three times in PBS (5 min) prior to imaging. Images were acquired using either a 100X lens on imaging system 2 (endothelial patches) or a 60X 1.0 NA water dipping lens on imaging system 1 (*en face* arterial blood vessels) with 5% LED light (100 ms exposure). 100 images were acquired and averaged for each channel. Images are presented as an average intensity projection with a Gaussian blur (*σ* = 1) applied.

### 2.12 Solutions and Drugs

Cal520/AM was obtained from Abcam. Pluronic F‐127, calcein/AM, FluoVolt Membrane Potential Kit, secondary antibodies: donkey anti‐goat 488, donkey anti‐goat 568, donkey anti‐rabbit 555, donkey‐anti rabbit 647, donkey anti‐mouse 488 were obtained from Invitrogen. Acetylcholine, NaCl, MOPS, KCl, NaH2PO4, Glucose, EDTA, MgCl, EGTA, U73122, sulforhodamine B, LY294002, wortmannin, Gap27, saponin were obtained from Sigma Aldrich. FR900359 was obtained from Cambridge Bioscience Limited. NF 449, BAPTA/AM, Gallein, YM254890, were obtained from Tocris. Ins(1,4,5) P3‐caged IP_3_ was obtained from SiChem. All solutions were prepared fresh each day.

### 2.13 Data and Statistical Analysis

Summarized data are presented as individual data points, and matched experiments are indicated graphically by lines linking data points; “*n*” refers to the number of animals. Data were compared using various statistical tests, as indicated in the corresponding text and/or figure legends. A post hoc test was only performed if *F* was significant and there was no variance inhomogeneity. All statistical analyses were performed using GraphPad Prism, version 6.0 (GraphPad Software). A *p* value < 0.05 was accepted as statistically significant. All tests, *n* numbers and significance values are noted in the figure legends.

## 3 Results

**FIGURE 1:**
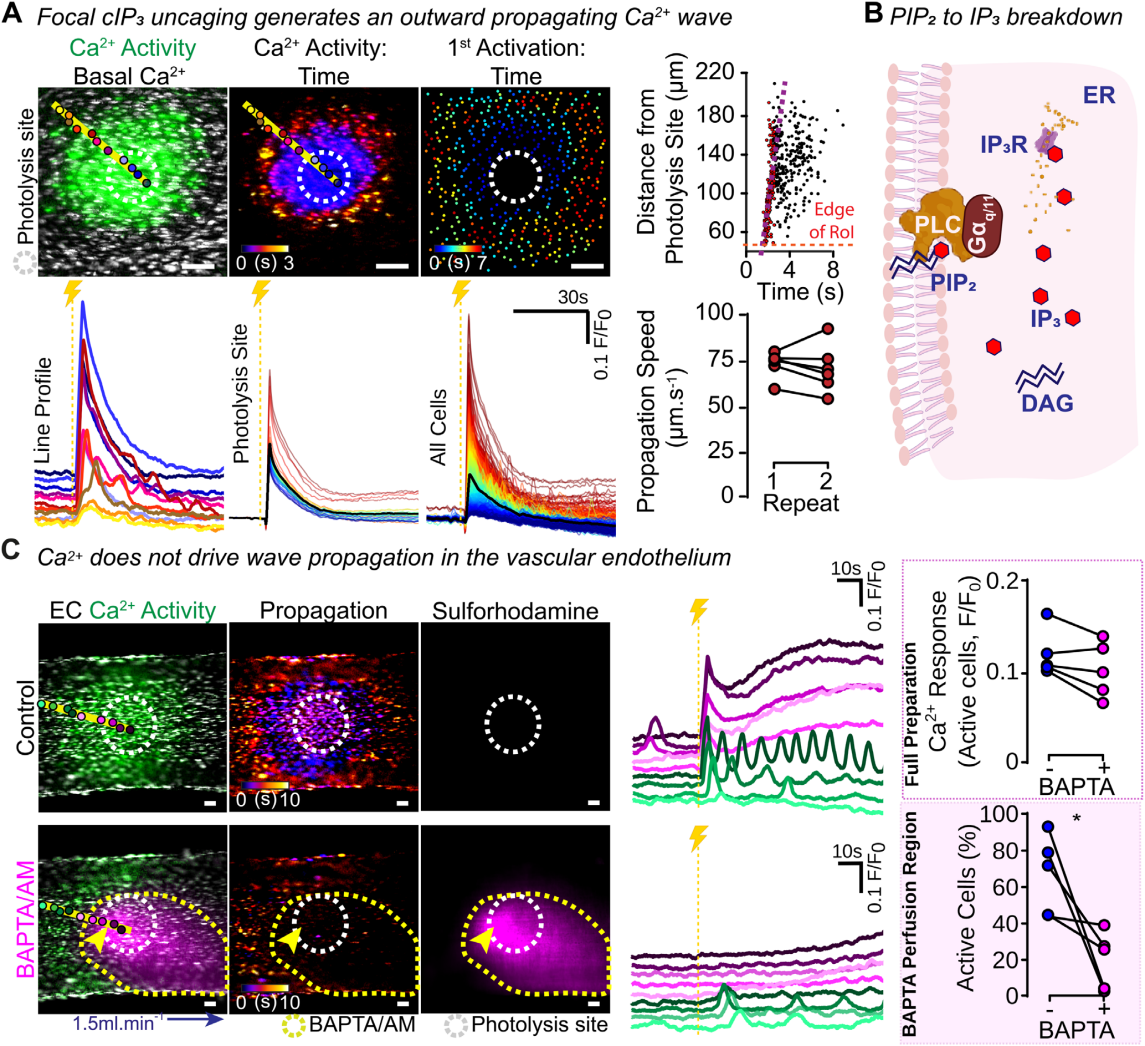
Focal release of caged IP_3_ in the vascular endothelium generates an IP_3_‐dependent radially propagating Ca^2+^ wave. (A) Representative live‐cell images of endothelial fields in an *en face* blood vessel loaded with Cal520/AM (5 μM) and cIP_3_ (5 μM) show basal Ca^2+^ levels (gray) and cIP_3_‐evoked Ca^2+^ activity (green). Color‐coded time maps of Ca^2+^ propagation for the first 3 s post activation and the first 7 s are shown. Individual traces from cells within the endothelial field are shown using three different methods. Traces are shown from cells underlying the marked circles along the yellow line (line analysis; separated vertically for ease of identification; color corresponding to the identification circles), within the photolysis site, or from every cell in the field (overlaid; color‐coded for F/F_0_ intensity, from red; high to blue; low). Plotting cell distance from the photolysis site against activation time confirms radial signal propagation. Red points are signals from within the photolysis site. The average Ca^2+^ propagation speed is shown for two repeats, with no significant difference between trials (*p* > 0.05) using a paired Student's *t*‐test. (B) Schematic showing activation of phospholipase C (PLC) to mediate breakdown of PIP_2_, generate IP_3_, and trigger Ca^2+^ release from the endoplasmic reticulum (ER). (C) Endothelial cells loaded with Cal520/AM (5 μM) and cIP_3_ (5 μM) show basal Ca^2+^ levels (gray) and cIP_3_‐evoked Ca^2+^ activity (green) overlaid, color‐coded time maps of Ca^2+^ propagation 10 s post uncaging, and the sulforhodamine B signal designating the BAPTA/AM region of influence (magenta), all in the presence of counter‐propagating PSS flow (1.5 mL.min^−1^; blue arrow). Images are shown before and after focal BAPTA/AM (30 μM, 15 min, yellow arrow: Puffer location) application using pressure ejection from a puffer pipette to a preselected endothelial region (yellow dashed line delineating magenta application region). Individual traces from cells within the endothelial field are shown from cells underlying the marked circles along the yellow line (separated vertically for ease of identification; color corresponding to the identification circles). Summary data show that focal buffering of Ca^2+^ with BAPTA/AM does not change the Ca^2+^ response in activated cells across the vessel, but significantly reduces the number of cells activated within the BAPTA/AM puffing region (purple‐shaded box, *n* = 5). Yellow dashed line with lightning bolt indicates photolysis. Scale bars = 50 μm. All summary data are matched; *Indicates statistical significance (*p* < 0.05) using a paired Student's *t*‐test.

Endothelial cells line the lumen of arteries and maintain tight cell–cell contact, regulating cell–cell signaling pathways. A dominant form of signaling control for endothelial cells is mediated through IP_3_‐activated release of Ca^2+^ from internal stores within the endoplasmic reticulum. To explore the mechanisms responsible for intercellular communication, experiments were conducted to visualize propagated Ca^2+^ responses in intact blood vessels. Intracellular Ca^2+^ signaling was initiated by local activation of IP_3_Rs in selected cells. This was achieved through the photolysis of a photolabile form of the inositide (caged IP_3_; cIP_3_). Upon photolysis, cIP_3_ triggered a rapid rise in intracellular Ca^2+^ at the activation site, which then propagated radially outwards from the circular uncaging site (Figure 1A). The speed of Ca^2+^ wave was reproducible across experimental repeats (Repeat 1: 73 ± 7 μm.s^−1^, Repeat 2: 70 ± 13 μm.s^−1^; Figure 1A).

Since the propagated response was visualized using the Ca^2+^ indicator, Cal520/AM, we initially hypothesized that Ca^2+^ itself drove the propagation across endothelial cells. To test whether Ca^2+^ was necessary for cIP_3_‐evoked Ca^2+^ wave propagation, the acetoxymethyl ester (AM) form of the Ca^2+^ chelator, BAPTA, was used to prevent the Ca^2+^ increase from occurring in a selected population of cells to reveal if the Ca^2+^ wave propagated beyond that region. BAPTA/AM was applied continuously for 15 min via a pressure ejection pipette, followed by 15 min for enzymatic hydrolysis of the AM group (Figure 1C). BAPTA/AM was applied to the entire photolysis site and the surrounding cells to ensure that cytoplasmic Ca^2+^ was buffered in all cells where cIP_3_ photolysis occurred. The precise region of BAPTA/AM application was visualized using the fluorophore sulforhodamine, which was also present in the puffer pipette. We have previously shown that the application of sulforhodamine onto *en face* blood vessels via pressure ejection does not elicit a Ca^2+^ response [10].

Following BAPTA/AM, photolysis of cIP_3_ did not evoke a Ca^2+^ response at the activation site, and there was a significant reduction in the number of activated cells within the BAPTA/AM puffing region (Figure 1C). However, outside the Ca^2+^‐buffered region, an outwardly propagating Ca^2+^ response occurred, generating a Ca^2+^ increase equivalent to that seen in the control recording (Figure 1C, Video S1). These findings suggest that an increase in IP_3_ is sufficient to drive wave propagation, and that Ca^2+^ itself is not required for signal propagation to occur.

### 3.1 Regenerative IP_3_ Production Drives Ca^2+^ Wave Propagation

**FIGURE 2:**
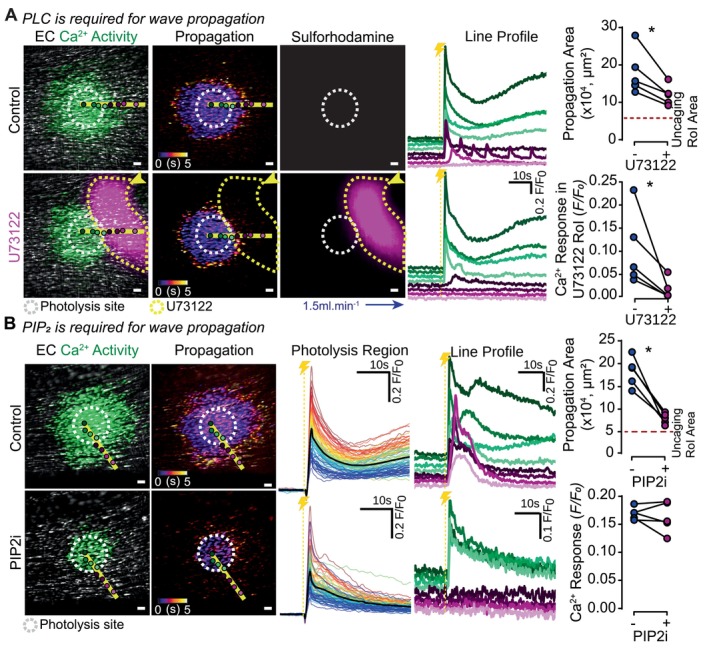
IP_3_‐mediated IP_3_ production, not Ca^2+^, is required for endothelial Ca^2+^ wave propagation. (A) Representative live‐cell images of an endothelial field loaded with Cal520/AM (5 μM) and cIP_3_ (5 μM) show basal Ca^2+^ levels (gray) and cIP_3_‐evoked Ca^2+^ activity (green), color‐coded time maps of Ca^2+^ propagation over the first 5 s post activation, and sulforhodamine B (1 μM) signal designating the U73122 region of influence. All images were acquired in the presence of PSS flow (1.5 mL.min^−1^; blue arrow). Images are shown before and after focal U73122 (10 μM, 15 min, yellow arrow: Puffer location) application using pressure ejection from a puffer pipette to a preselected endothelial region (yellow dashed line delineating magenta application region). Individual traces from cells within the endothelial field are shown from cells underlying the marked circles along the yellow line profile (separated vertically for ease of identification; color corresponding to the identification circles). Summary data show focal PLC inhibition with U73122 significantly reduces the propagation area and the Ca^2+^ response within the region of PLC inhibition (*n* = 5). (B) Representative live‐cell images of an endothelial field loaded with Cal520/AM (5 μM) and cIP_3_ (5 μM) show basal Ca^2+^ levels (gray) and cIP_3_‐evoked Ca^2+^ activity (green) and color‐coded time maps of Ca^2+^ propagation over the first 5 s post activation. Images are shown before and after global inhibition of PIP_2_ synthesis (300 μM LY294002 and 50 μM wortmannin, 30 min). Individual traces from cells within the endothelial field are shown from cells within the photolysis site (overlaid; color‐coded for F/F_0_ intensity, from red; high to blue; low) or underlying the marked circles along the yellow line profile (separated vertically for ease of identification; color corresponding to the identification circles). Yellow dashed line with lightning bolt indicates photolysis. Summary data show that blocking PIP_2_ generation with LY294002 and wortmannin reduces the Ca^2+^ wave propagation area without reducing the average Ca^2+^ response in the photolysis site (*n* = 5). All summary data are matched; *Indicates statistical significance (*p* < 0.05) using a paired Student's *t*‐test. Scale bars = 50 μm.

IP_3_ generation occurs via activation of phospholipase C (PLC) in the cell membrane, leading to the hydrolysis of phosphatidylinositol‐4,5‐bisphosphate (PIP_2_) to IP_3_ (Figure 1B). To investigate whether regenerative IP_3_ production was required for endothelial Ca^2+^ wave propagation to occur, we used the PLC inhibitor U73122 (10 μM, 15 min) to focally block PLC activity in a selected population of cells and prevent PIP_2_ degradation to IP_3_. U73122 was applied locally via pressure ejection in the direction of PSS flow, to a region of cells adjacent to the propagation region, to inhibit PLC (Figure 2A). In the presence of U73122, the propagated Ca^2+^ response was blocked within the U73122‐incubated areas, resulting in a significant reduction in the area of propagation (Figure 2A). The number of active cells and the Ca^2+^ response, within the U73122‐incubated region, were also significantly decreased (Figure 2A). This finding suggests that regenerative IP_3_ production plays a crucial role in Ca^2+^ wave propagation.

To validate the role of regenerative IP_3_ production, we inhibited another step in the pathway, PIP_2_ generation, using phosphatidylinositol 4‐kinase (PI4K) inhibitors LY294002 (300 μM) and wortmannin (50 μM) [19, 20, 21, 22]. Incubation of PI4K inhibitors for 30 min significantly reduced the propagation area, restricting it to just beyond the photolysis site, without altering the average cIP_3_‐evoked Ca^2+^ response in the photolysis site (Figure 2B).

Collectively, these experiments demonstrate that Ca^2+^ does not drive rapid outward wave propagation in mesenteric artery endothelial cells. Instead, regenerative IP_3_ production is the key mechanism driving Ca^2+^ wave propagation.

### 3.2 IP_3_ Diffusion Through Gap Junctions Does Not Influence Ca^2+^ Wave Propagation

The question now arises, what triggers regenerative IP_3_ production in cells outside of the photolysis region? A prevalent hypothesis for intercellular Ca^2+^ wave propagation in the endothelium is that gap junctions facilitate movement of IP_3_, as a second messenger, operating between cells. Therefore, we next tested the role of gap junctions in wave propagation.

IP_3_ is a trivalent anion that is proposed to diffuse passively between cells through gap junctions, driven by the electrochemical gradient operating on the inositide. The increase in IP_3_ triggers a rise in intracellular Ca^2+^, which activates Ca^2+^‐dependent K^+^ channels, leading to hyperpolarization of the activated cells. This hyperpolarization will increase the electrochemical gradient for IP_3_ movement, thereby facilitating its flux between cells [23]. A further increase in the electrochemical gradient can be achieved by selectively depolarizing those cells that are directly coupled to those releasing IP_3_. If gap junctions allow IP_3_ passage, as is commonly hypothesized [8, 9], depolarizing adjacent electrically‐coupled cells would increase the driving force for IP_3_ entry into those cells, promoting more extensive wave propagation.

**FIGURE 3:**
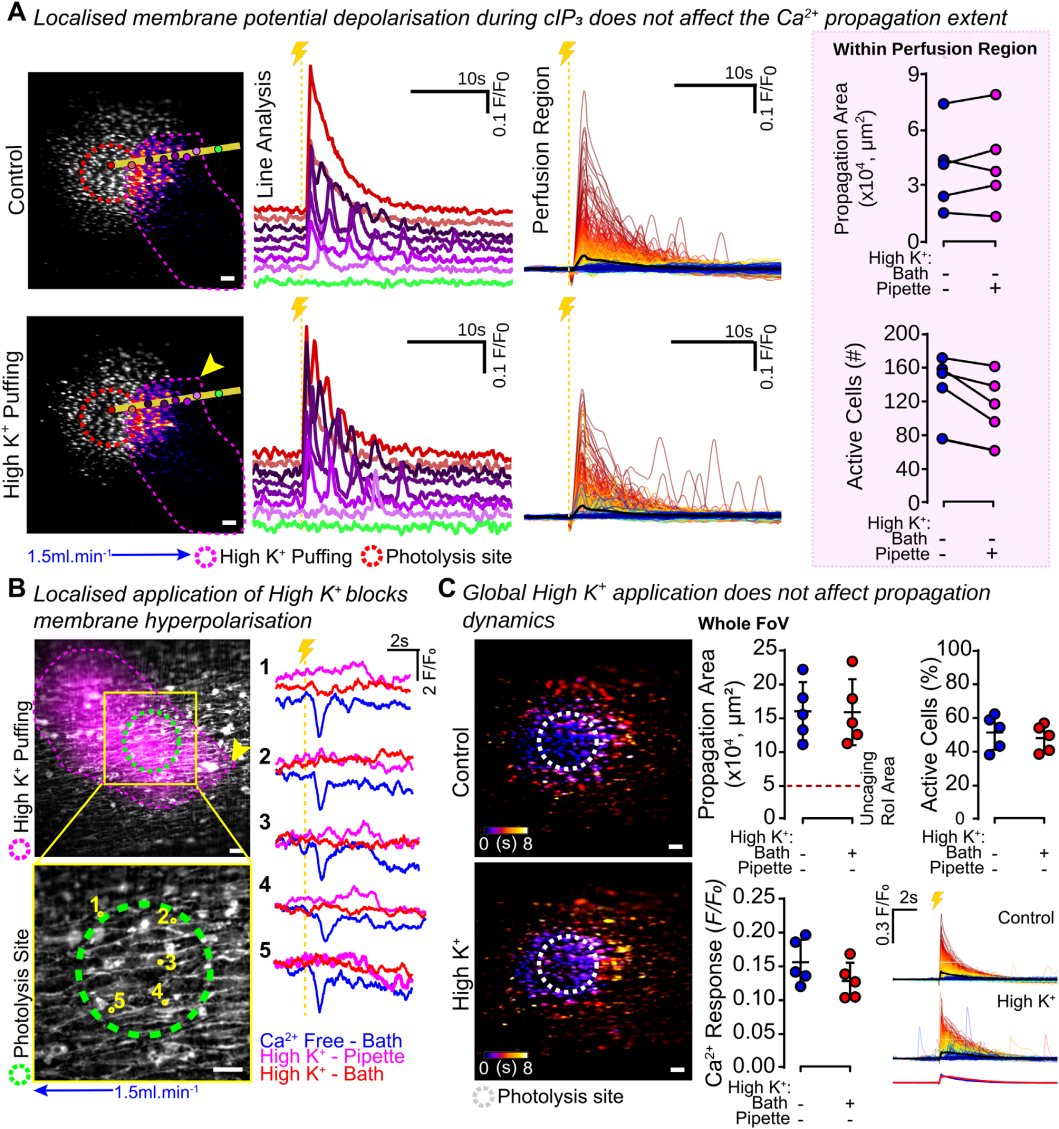
Localized membrane potential depolarization does not alter Ca^2+^ wave propagation. (A) Representative live‐cell images of an endothelial field loaded with Cal520/AM (5 μM) and cIP_3_ (5 μM) show cIP_3_‐evoked Ca^2+^ activity (gray) over the first 5 s postactivation, under control conditions or after focal perfusion of High K^+^ (134 mM) PSS using pressure ejection from a puffer pipette to a preselected endothelial region at the outer extent of signal propagation (purple dashed line and FIRE LUT delineating magenta application region; yellow arrow: Puffer location). All images were acquired in the presence of Ca^2+^ free PSS flow (1.5 mL.min^−1^; blue arrow). Individual traces from cells within the endothelial field are shown from cells within the High K^+^ perfusion region (overlaid; color‐coded for F/F_0_ intensity, from red; high to blue; low) or underlying the marked circles along the yellow line profile (separated vertically for ease of identification; color corresponding to the identification circles). Yellow dashed line with lightning bolt indicated photolysis. Summary data showing that within the High K^+^ perfusion region, focal membrane potential depolarization does not change the propagation area, nor the number of active cells (*n* = 5, matched data). (B) Image of an endothelial field stained with FluoVolt (5 min incubation, gray). Signal traces are shown from 5 locations (numbered yellow labels) under control conditions (counter propagating Ca^2+^ free PSS; blue line), high K^+^ PSS puffed onto the vessel using pressure ejection from a puffer pipette in the presence of counter propagating Ca^2+^ free PSS (magenta application region; magenta line), and global counter propagating high K^+^ PSS (red line) in the entire bath. (C) Color‐coded time maps of cIP_3_‐evoked Ca^2+^ propagation over the first 5 s postactivation under control conditions (Ca^2+^ free PSS) or after global high K^+^ (134 mM) PSS. Summary data show that high K^+^ (134 mM) PSS does not affect the propagation area, the Ca^2+^ response nor the number of cells activated (*n* = 5). Representative traces of Ca^2+^ activity from each cell within the field of view are shown; the average signal is represented by a thick line and shown overlaid. Perfusion region‐of‐influence was determined using sulforhodamine B (1 μM) signal. *Indicates statistical significance (*p* < 0.05) using a paired Student's *t*‐test. All High K^+^ PSS is also Ca^2+^‐free to avoid contraction of arteries. Scale bars = 50 μm.

To test this hypothesis, we depolarized a preselected population of endothelial cells adjacent to the outer edge of the IP_3_ photolysis region using high K^+^ PSS (134 mM; E_K_ ~ −1 mV), applied focally via a pressure ejection pipette (Figure 3A). These experiments were conducted in Ca^2+^‐free PSS to prevent smooth muscle cell contraction, and the region of high K^+^ PSS application was identified using the fluorophore sulforhodamine, which was also present in the puffer pipette.

There was no significant difference between either the F/F_0_ responses in cells along the line profile drawn, in the average propagation area within the High K^+^‐perfused region, or between the active cell number in the High K^+^‐perfused region (Figure 3A). These results challenge the expectation that gap junctions, by allowing the passive flux of IP_3_ between cells, are primarily responsible for mediating wave propagation (Figure 3A, Video S2).

To ensure that membrane potential was depolarized as expected, we first checked that high K^+^ PSS blocked cIP_3_‐evoked hyperpolarization. In these experiments, the endothelium was dual‐loaded with the membrane potential indicator FluoVolt and cIP_3_. Photolysis of cIP_3_ (once again in Ca^2+^‐free PSS) was recorded with either localized high K^+^ PSS perfused across the photolysis region or present in the entire bath (Figure 3B). High K^+^ PSS in the puffer pipette or throughout the bath each blocked cIP_3_‐evoked hyperpolarization, as expected from the high K^+^‐evoked shift in E_K_ (Figure 3B).

In a separate series of experiments, puffing of high K^+^ PSS across the entire vessel did not affect the propagation area, the Ca^2+^ response nor the number of active cells when compared to control responses, evoked in Ca^2+^ free PSS (Figure 3C). This experiment establishes that the store content and responsiveness to IP_3_ were unchanged by high K^+^ PSS over the time course of the experiments.

As an additional control, to test that each artery used responded appropriately, high K^+^ PSS puffed onto the *en face* blood vessels caused a rapid contraction, as expected, when Ca^2+^ was present in the bath PSS (Figure S1).

These results suggest that gap junctions may play a limited role in the movement of IP_3_ during Ca^2+^ wave propagation.

The effects of gap junction blockers on wave propagation were also examined in a subsequent series of experiments. While there are many effective pharmacological gap junction blockers, we have previously shown that the widely used inhibitors carbonoxolone and 18β‐glycyrrhetinic acid inhibit IP_3_R activity and depolarize mitochondria [16], making these inappropriate to use in endothelial Ca^2+^ wave propagation studies. Another widely used inhibitor, 18α‐glycyrrhetinic acid, effectively blocks diffusion through gap junctions, though it does not affect cIP_3_‐evoked propagation extent [10].

Gap27 is a peptide derived from connexin 43, a connexin subtype that is widely found in the mesenteric vasculature [24], and as such is a selective gap junction blocker [25]. We tested whether or not Gap27 altered wave propagation dynamics in endothelial cells. Gap27 (300 μM, 30 min) did not alter the average Ca^2+^ response, nor the propagation area of the cIP_3_‐evoked Ca^2+^ wave (Figure S2A). To verify that gap junctions were being blocked effectively by Gap27 at the concentration and incubation time used, “Fluorescence recovery after photobleaching” (FRAP) experiments were performed in *en face* blood vessels stained with the fluorophore calcein/AM (MW_calcein_ = 620 Da; MW_IP3_ = 420 Da). The diffusion of calcein to neighboring cells after photobleaching was recorded. In controls, full recovery occurred after 1 h, however this recovery was blocked in the presence of Gap27, suggesting that the blocking of gap junctions occurred (Figure S2B).

This evidence, taken together, suggests that neither gap junctions nor transcellular diffusion plays a role in the rapid, radial IP_3_‐evoked Ca^2+^ wave propagation.

### 3.3 Gα_q/11_ GPCRs Are Involved in Regenerative IP_3_ Production

The results presented suggest that PLC‐mediated regenerative IP_3_ production drives wave propagation. It follows that part of an intercell communication bridge may therefore involve IP_3_ in one cell, resulting in G protein‐mediated activation of PLC in a neighbor to evoke the breakdown of PIP_2_ into IP_3_ in the cells outside the photolysis site.

**FIGURE 4:**
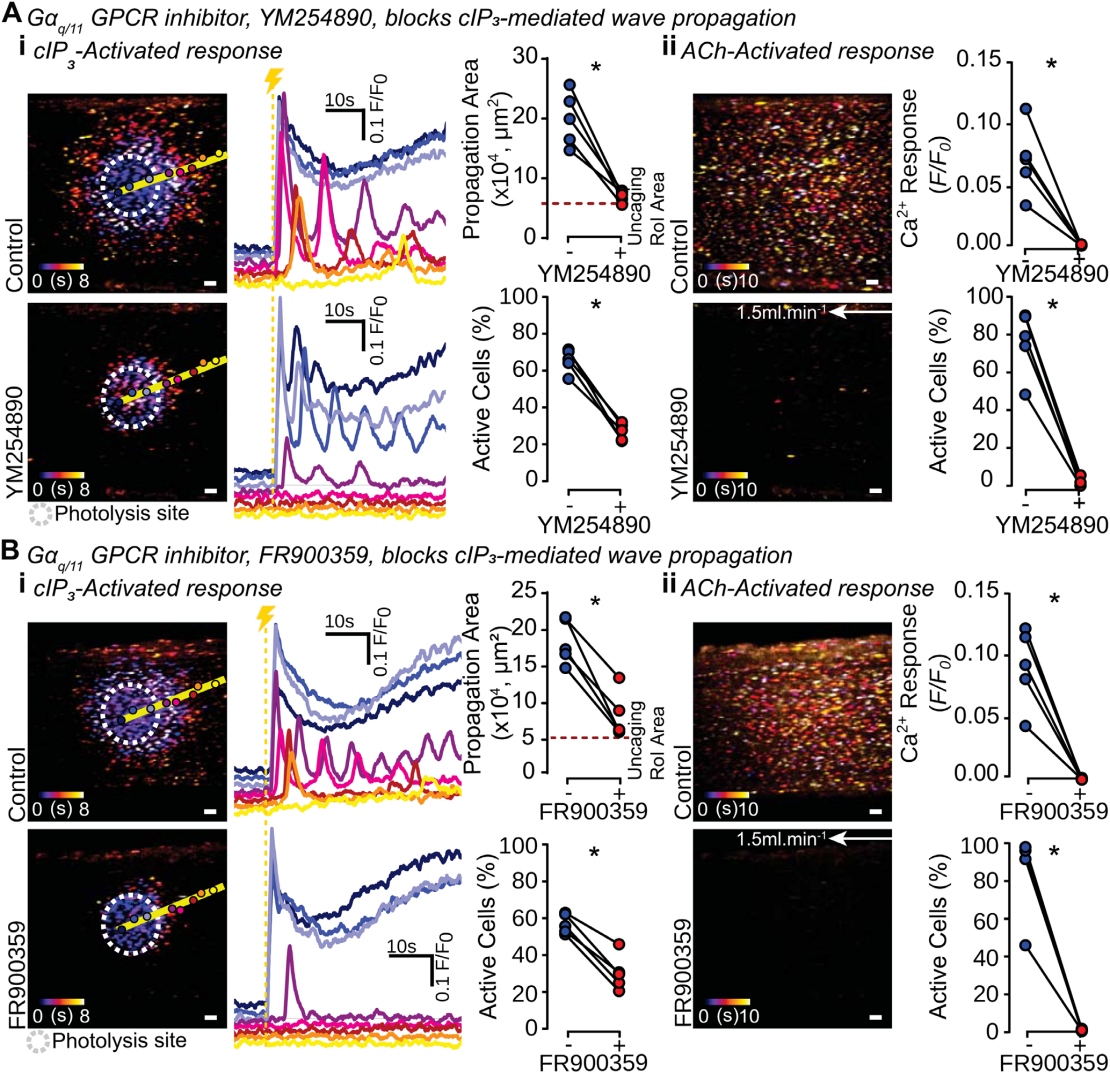
Gα_q/11_ GPCRs are required for regenerative IP_3_‐mediated IP_3_ production. Representative live‐cell images of an endothelial field loaded with Cal520/AM (5 μM) and cIP_3_ (5 μM) show basal Ca^2+^ levels (gray) and color‐coded time maps of Ca^2+^ propagation arising from (i) cIP_3_‐generated and (ii) ACh (100 nM)‐generated activation. Images are shown under control conditions or after Gα_q/11_ inhibition across the entire vessel using (A) YM254890 (1 μM, 30 min) or (B) FR900359 (5 μM, 30 min). Individual traces from cells within the endothelial field are shown from cells underlying the marked circles along the yellow line profile (separated vertically for ease of identification; color corresponding to the identification circles). A yellow dashed line with a lightning bolt indicates photolysis. Summary data show that blocking Gα_q/11_ GPCR activity with either YM254890 or FR900359 reduces the cIP_3_‐evoked propagation area and number of activated cells (*n* = 5). A significant decrease in the number of ACh‐evoked activated cells and the Ca^2+^ response in YM254890‐incubated or FR900359‐incubated vessels (*n* = 5) was seen. Summary data are all matched; *Indicates statistical significance (*p* < 0.05) using a paired Student's *t*‐test. Scale bars = 50 μm.

To test this possibility, a selective inhibitor of Gα_q_‐mediated signaling (YM254890; 1 μM, 30 min) [26] was first used. YM254890 blocked propagation of cIP_3_‐evoked Ca^2+^ waves, significantly reducing the number of active cells while having no effect on the cIP_3_‐evoked Ca^2+^ response within the photolysis region (Figure 4Ai, Video S3).

Gα_q/11_ GPCRs are activated by the M3 muscarinic receptor; therefore, confirmation of YM254890 inhibitory activity was achieved using the M3 agonist ACh. ACh controls, performed in the same arterial blood vessels, were blocked when Gα_q/11_ GPCR activity was inhibited by YM254890, causing a significant decrease in both the average Ca^2+^ response and the percentage of ACh‐activated cells (Figure 4Aii). These results suggest that Gα_q/11_ contributes to Ca^2+^ wave propagation.

A second Gα_q/11_ inhibitor (FR900359; 5 μM, 30 min) [27] was used to confirm these findings. The extent of cIP_3_‐evoked propagation and the number of active cells were significantly decreased in the presence of FR900359 while the cIP_3_‐evoked Ca^2+^ increase in the photolysis site was unaltered (Figure 4Bi). The ACh‐evoked Ca^2+^ response and the number of activated cells by ACh were significantly decreased by FR900359, confirming the inhibition of Gα_q_ G proteins. Taken together, these data (Figure 4Bii) suggest a novel role for Gα_q/11_ GPCRs as part of the transcellular communication bridge, facilitating regenerative IP_3_ production in wave propagation.

### 3.4 G_i/o_, G_s_ and Gβγ GPCRs Are Not Involved in Regenerative IP_3_ Production

PLCβ exists in four distinct isoforms (1–4), which have each been shown to respond to stimuli from either Gα_q_ GPCRs or G_i_‐ and Gβγ‐GPCRs (from G_i_‐Gβγ heterodimers) [28]. G_i_ or Gβγ subunits may also contribute to the regenerative IP_3_ production pathway via activation of distinct PLC isoforms. To test this, we inhibited the signaling of the Gβγ and G_i/o_ subunits.

**FIGURE 5:**
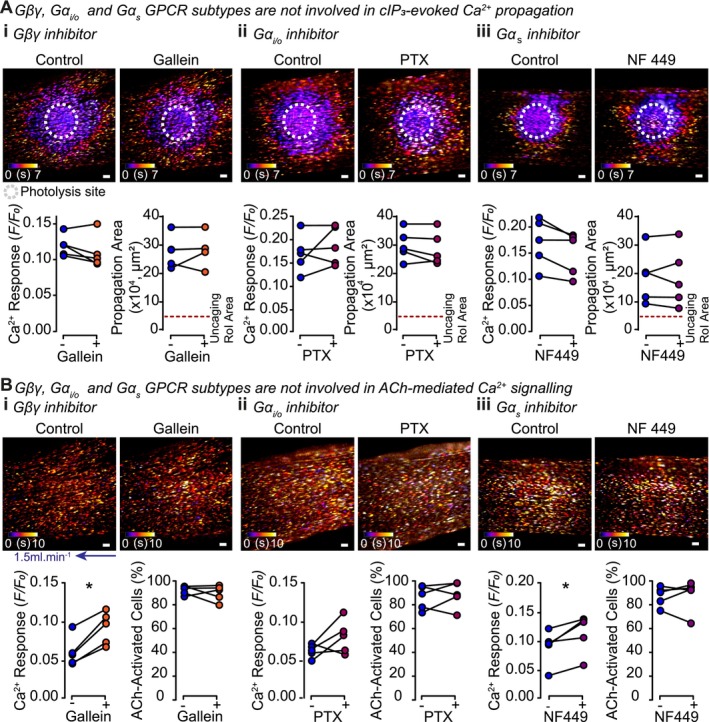
Gβγ, Gα_i/o_, and Gα_s_ GPCRs are not involved in regenerative IP_3_‐mediated IP_3_ production. Representative live‐cell images of an endothelial field loaded with Cal520/AM (5 μM) and cIP_3_ (5 μM) show color‐coded time maps of (A) cIP_3_‐evoked or (B) ACh‐evoked Ca^2+^ propagation, under control conditions and after global (i) Gβγ inhibition using Gallein (5 μM, 30 min), (ii) Gα_i/o_ inhibition using Pretussis toxin (PTX 100 ng.mL^−1^, 4 h), or (iii) Gα_s_ inhibition using NF 449 (1 μM, 30 min). Summary data show that blocking Gβγ, Gα_i/o_, or Gα_s_ GPCR activity with either Gallein, PTX, or NF 449, respectively, does not alter the cIP_3_‐activated Ca^2+^ response, nor does it reduce the propagation area (*n* = 5). In ACh controls, summary data show no alteration in the number of cells activated, and an increase in the ACh‐activated Ca^2+^ response (*n* = 5). Summary data are all matched; *Indicates statistical significance (*p* < 0.05) using a paired Student's *t*‐test. Scale bars = 50 μm.

The Gβγ subunit signaling inhibitor, Gallein (5 μM, 30 min) [29] failed to alter the cIP_3_‐evoked propagation area or Ca^2+^ response (Figure 5Ai). Similarly, the G_i/o_ inhibitor pertussis toxin (PTX, 100 ng.ml^−1^, 4 h) [30] did not alter the cIP_3_‐activated Ca^2+^ response, nor the cIP_3_‐evoked propagation area (Figure 5Aii).

G_s_ proteins primarily act to increase cAMP levels within cells via stimulation of adenylyl cyclases [31]. Some studies have also shown that agonist binding to Gα_s_ stimulates PLC activity [32]. However, the G_s_ inhibitor NF 449 (1 μM, 30 min) [33] did not affect the cIP_3_‐evoked Ca^2+^ response, or the propagation area of the outwardly propagating wave (Figure 5Aiii).

Interestingly, whilst no changes were seen in the number of ACh‐activated cells in the presence of these Gβγ, G_i/o_, or G_s_ inhibitors, there was a consistent *increase* in ACh‐elicited Ca^2+^ responses in the presence of gallein and NF499 (Figure 5Bi–iii).

These results suggest that Gβγ, G_i/o_, and G_s_ G protein subfamilies do not play a role in the IP_3_‐evoked IP_3_ regenerative signaling cascade.

### 3.5 IP_3_ Receptors Are Located at the Plasma Membrane of Endothelial Cells

Collectively, the results thus far show that regenerative IP_3_ production, rather than Ca^2+^ itself, drives the propagation of Ca^2+^ waves, facilitated by transcellular activation of a Gα_q/11_‐coupled GPCR. These findings raise the question of how regenerative IP_3_ signaling is initiated and transmitted between neighboring cells during wave propagation?

A potential explanation is the presence of IP_3_Rs at the plasma membrane, which could act as transmembrane signaling hubs. These IP_3_Rs could activate a Gα_q/11_‐coupled GPCR on the adjacent receiving cell (without requiring ion permeation), triggering PLC‐mediated hydrolysis of PIP_2_ into IP_3_ and propagating the signal to neighboring cells.

**FIGURE 6:**
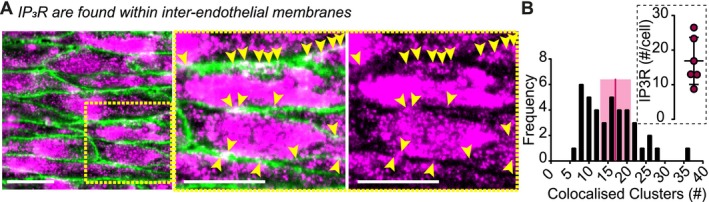
IP_3_R clusters are located near interendothelial membranes. (A) Immunofluorescence imaging of *en face* preparations with endothelial cells labeled for CD31/PECAM (green) and IP_3_Rs (magenta) shows colocalization (white) of IP_3_Rs with PECAM‐positive cell membrane regions. (B) Images were analyzed to count IP_3_R clusters located at the membrane (yellow arrowheads). A histogram displays the distribution of IP_3_R clusters counted across six endothelial cell patches, with the average cluster count overlaid. Scale bars = 20 μm.

To determine whether IP_3_Rs are found at or near the plasma membrane, endothelial cell patches were simultaneously stained using an anti‐PECAM (platelet endothelial cell adhesion molecule; CD31) antibody to label the plasma membrane, and an anti‐IP3R1 antibody to label IP_3_Rs (Figure 6). The results show that although IP_3_R distribution is primarily concentrated within the endothelial cell body, there is a peripheral, near‐membrane localization of IP_3_R clusters (arrowheads, Figure 6A). Using data from endothelial cell patches (*n* = 6 patches), an average of 17 ± 4 IP_3_R clusters per cell colocalized with CD31 staining (Figure 6B).

## 4 Discussion

In this study we show that elevated levels of IP_3_ in endothelial cells trigger a noncanonical inside‐out signaling mechanism to transcellularly activate Gα_q/11_‐coupled receptors in neighboring cells. Outside‐in GPCR activation then triggers canonical PLC‐mediated hydrolysis of PIP_2_ into IP_3_. This inside‐out outside‐in signaling mechanism drives a regenerative, IP_3_‐dependent signaling cascade between adjacent cells, facilitating intercellular communication. Importantly, the diffusion of Ca^2+^ or IP_3_ through gap junctions does not contribute significantly to this process.

In the endothelium, intercellular communication, over various time and spatial scales, is essential for coordinating physiological adjustments. Intercellular communication ensures precise, timely, and synchronized regulation of critical processes, such as vessel tone modulation, alterations in vascular permeability, and the regulation of immune responses.

Ca^2+^ is a key second messenger that acts both as a trigger for functional responses and as a potential mediator of intercellular signal transmission. However, understanding the specific role of Ca^2+^ and the precise mechanisms driving intercellular signal propagation within the endothelium remains a significant challenge. In the endothelium, the major source of Ca^2+^ in agonist activation is the release of the ion from the internal stores via the PLCβ‐PIP_2_‐IP_3_ pathway. PLCβ is the major G protein‐coupled enzyme that facilitates the breakdown of PIP_2_ into IP_3_ and DAG, leading to IP_3_‐IP_3_R binding at the ER and the release of Ca^2+^ into the cytoplasm. In this study, we induced the release of Ca^2+^ from internal stores in an IP_3_‐dependent, but PLC‐independent, manner. We then used pharmacological interventions to target each step of the PLC/Ca^2+^ signaling pathway to determine the mechanisms responsible for the propagation of intercellular cIP_3_‐evoked Ca^2+^ waves. The results demonstrate regenerative IP_3_‐mediated IP_3_ production, rather than the Ca^2+^ ion itself, drives Ca^2+^ wave propagation.

Two main lines of evidence support this conclusion. First, the Ca^2+^ chelator BAPTA/AM, selectively applied around the uncaging region, prevented Ca^2+^ from increasing after the local release of IP_3_. However, after the release of IP_3_, a Ca^2+^ wave propagated outside the BAPTA‐buffered region even though no IP_3_ had been released at these sites. These data show that IP_3_ generates a propagating Ca^2+^ wave but that the Ca^2+^ ion itself is not required for waves to propagate (Figure 1C). There was a slight reduction in the amplitude of the Ca^2+^ response in the presence of BAPTA/AM compared to the corresponding signal in the paired control, which could be attributed to low levels of BAPTA/AM diffusing beyond the perfusion area, leading to partial intracellular buffering. This finding is consistent with our previous work, which demonstrated that, although the photolabile Ca^2+^ chelator Diazo‐2/AM effectively blocked a cIP_3_‐induced Ca^2+^ increase at the photolysis site, a Ca^2+^ wave still propagated radially beyond the photolysis site [10].

Secondly, we show the cIP_3_‐evoked Ca^2+^ wave failed to propagate into regions where PLC had been selectively inhibited (Figure 2A) or where PI4K inhibitors [20] had been used to prevent the PIP_2_ hydrolysis necessary for IP_3_ production (Figure 2B). These results demonstrate that an IP_3_‐dependent mechanism activates PLC, leading to the production of IP_3_ in adjacent cells, thereby facilitating the propagation of regenerative Ca^2+^ waves. This process is independent of the rise in Ca^2+^.

Next, we sought to determine the nature of the link between an increase in IP_3_ and PLC activation. PLCβ activation and subsequent breakdown of PIP_2_ into IP_3_ is a process often initiated by GPCRs on the outer plasma membrane [34], therefore we hypothesized that a transcellular form of G protein signaling plays a role in this intercellular communication pathway.

GPCRs are membrane‐spanning receptors that customarily act as an interface between agonist‐binding at the cell surface and the resulting cascade of intracellular signaling that affects cell function. G proteins are heterotrimeric structures that contain three distinct subunits: membrane‐bound α and γ subunits, and β subunits that exist as a dimer with γ. In the inactive state, GDP is bound to the Gα subunit. Agonist‐binding causes a conformational change in the GPCR, releasing GDP from Gα and allowing GTP to bind, releasing the Gα‐GTP and Gβγ subunits to effect downstream signaling [35].

There are four prominent classes of G protein families: Gα_q/11_, Gα_i/o_, Gα_s_ and Gβγ. Since Gα_q/11_ is the primary G protein subfamily that activates PLCβ at the plasma membrane [2, 3], it was tempting to speculate that it may be involved in regenerative IP_3_‐mediated wave propagation. Using the Gα_q/11_ inhibitors YM254890 and FR900359, we show for the first time that regenerative IP_3_‐mediated Ca^2+^ wave propagation is dependent on transcellular Gα_q/11_ signaling. YM254890 [26, 36] and FR900359 [27, 37] each block the exchange of GDP for GTP during Gα_q/11_ activation by binding the cleft between two interdomain linkers connecting the Gα_q_ GTPase and helical domains required for nucleotide access to the binding site. Previous studies found no off‐target Gα_i_–coupled receptor activity of YM254890, nor any indication that PLCβ and its downstream signaling pathway were targeted [38]. Effective inhibition of Gα_q/11_‐mediated activity via both drugs was confirmed by successful inhibition of ACh‐mediated M3‐receptor Gα_q/11_ activation (Figure 4). Furthermore, FR900359 Gα_q/11_ inhibition has also been confirmed by reversing phenylephrine‐mediated α1‐adrenergic receptor–dependent constriction of tail arteries [37]. Neither YM254890 nor FR900359 affected the amplitude of the IP_3_‐evoked Ca^2+^ response when the receptor was directly activated by IP_3_, indicating that the inhibitors did not have any off‐target binding downstream of the membrane GPCR signaling. Since the G protein inhibitors (YM254890 and FR900359) prevent wave propagation evoked by direct activation of IP_3_R, we show for the first time that Gα_q/11_ release and activation of PLC are required for intercellular signal propagation to occur. Our results also suggest that none of Gα_i/o_, Gα_s_ or Gβγ contributes to IP_3_‐mediated Ca^2+^ wave propagation.

**FIGURE 7:**
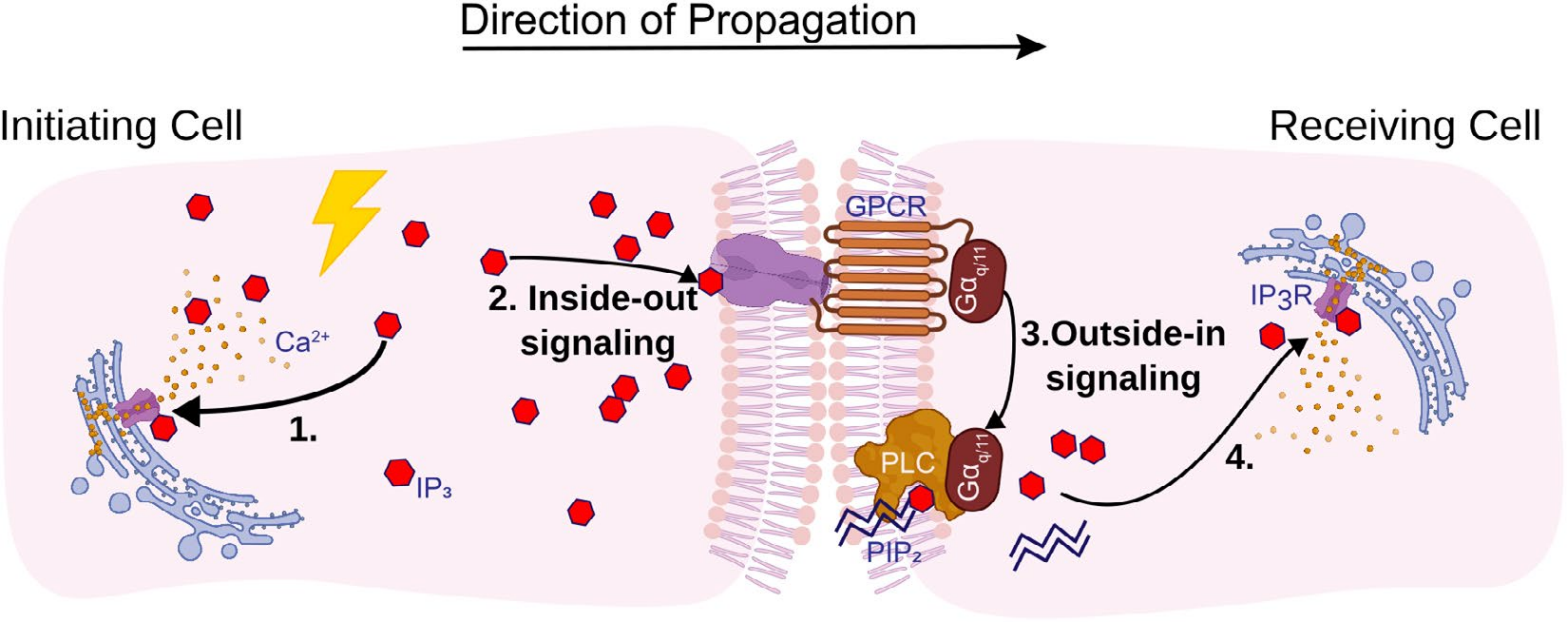
Summary of the proposed IP_3_‐mediated signaling pathway that maintains intercellular communication in the vascular endothelium. (1) IP_3_ activates IP_3_R on the endoplasmic reticulum within the initiating cell, releasing Ca^2+^ into the cytoplasm. (2) The signal is passed through to the receiving cell via a transmembrane signaling unit, in a process of “Inside‐out signaling.” Since IP_3_ is required for signal transmission, we hypothesize that this is mediated by pore dead IP_3_Rs, clustered at the membrane. (3) This inside‐out signal activates a GPCR on the receiving cell membrane, in an “Outside‐in signaling” process, releasing the Gα_q/11_ subunit to bind to PLC, allowing it to mediate the breakdown of PIP_2_ into free IP_3_ and DAG in the receiving cell cytoplasm. This is regenerative IP_3_ production. (4) Newly‐generated IP_3_ then binds the IP_3_R on the ER in the receiving cells, releasing Ca^2+^ and starting the regenerative process over again to propagate the signal along the vascular endothelium.

Whilst the present experiments provide a key insight into the signaling pathway, they do not elucidate the whole picture. Our results show that IP_3_ produced in one cell can trigger IP_3_ and Ca^2+^ release in neighboring cells, leading to the propagation of Ca^2+^ waves across the endothelium. This process therefore relies on both inside‐out and outside‐in signaling. While the precise mechanisms are unclear, our findings suggest that IP_3_ activates an IP_3_ receptor at the plasma membrane of the initiating cell (inside‐out signaling), which then directly or indirectly engages a Gα_q/11_‐linked GPCR on an adjacent cell. This GPCR activation initiates outside‐in signaling, resulting in further IP_3_ production from PLC‐PIP_2_ and continuing the propagation cycle. A membrane‐spanning unit on the initiating cell must exist to activate the Gα_q/11_‐coupled receptor on the receiving cell. This proposal is summarized in Figure 7, where an increase in IP_3_ in the initiating cell causes Ca^2+^ release from the internal stores (1). IP_3_ then triggers an event on the initiating cell plasma membrane (2) which leads to the activation of a Gα_q/11_‐coupled receptor on the receiving cell membrane (3). The Gα_q/11_ subunit is released, activating PLC‐mediated breakdown of PIP_2_ into IP_3_ and DAG, with IP_3_ binding to IP_3_R and releasing Ca^2+^ into the cytoplasm of the receiving cell (4). However, a critical question remains: how are signals transmitted between adjacent cells?

One possibility is via gap junctions. Gap junctions consist of linked connexin hemichannels on adjacent cell boundaries which connect the cytoplasm of each cell via a transmembrane pore [39]. Gap junctions have been proposed to play a role in intercellular communication in many cell types [40, 41, 42, 43], including the vascular endothelium [44, 45, 46, 47]. It has been widely reported that endothelial Ca^2+^ wave propagation is facilitated by diffusion of IP_3_ through gap junctions, causing an increase in intracellular Ca^2+^ by binding to IP_3_Rs and initiating further Ca^2+^‐induced IP_3_ production [5, 9, 48]. While we also clearly see diffusion through gap junctions (Figure S2 [10]), the time scale required for this signal propagation to occur does not align with the speed of cIP_3_‐evoked radial propagation. Photolysis of 5 μM of caged IP_3_ generates 0.5 μM IP_3_, which is hydrolyzed at ~0.3 μM.s^−1^. IP_3_ itself has a short half‐time (~1 s) [49] and a predicted range of action of 14 μm [50]. On the other hand, the measured propagated Ca^2+^ waves last for tens of seconds and extend hundreds of microns. This evidence suggests that diffusion of IP_3_ itself is unlikely to account for wave propagation.

Further evidence that challenges the involvement of IP_3_ diffusion between cells is provided by the observation that changes in the plasma transmembrane potentials do not affect Ca^2+^ wave propagation. IP_3_ is a trivalent anion, and any flux between cells via gap junctions will be influenced by the electrochemical driving force acting on the inositide. Following a localized increase in IP_3_, depolarization of neighboring electrically coupled cells will enhance the electrochemical driving force for IP_3_ flux between cells. This depolarization should thereby promote more extensive wave propagation. However, no increase in wave propagation was observed. In these experiments, a high K^+^ (Ca^2+^ free) PSS was applied to cells adjacent to the photolysis site in which IP_3_ was released (Figure 3). The high K^+^ PSS will produce around a 40 mV depolarization and clamp the membrane potential to ~ −1 mV. This membrane potential change is equivalent, in terms of the change in driving force acting on IP_3_, to a 125‐fold increase in the concentration of IP_3_ in the activated cells. Despite the increased driving force, no change in wave propagation occurred. These experiments suggest that diffusion of uncaged IP_3_ between cells via gap junctions is unlikely to drive wave propagation.

The role of gap junctions in permitting diffusion of second messengers is often examined using pharmacological inhibitors. However, many of these inhibitors have off‐target effects, so they must be used with caution when investigating intercellular Ca^2+^ wave propagation. 18β‐glycyrrhetinic acid and carbenoxolone block IP_3_R activity [16], and alcohol‐based blockers such as octanol and heptanol similarly dampen IP_3_‐mediated Ca^2+^ release and capacitative Ca^2+^ entry [51]. These effects of the inhibitors will block intercellular Ca^2+^ propagation independently of the contribution of gap junctions to the process. Attention should therefore be given to ensuring an unaltered initial Ca^2+^ response when interpreting the reduction of intercellular Ca^2+^ waves by gap junction inhibitors.

In the present study, we show that Gap27, a specific connexin 43 mimic that blocks gap junction function [52], inhibits fluorophore diffusion after FRAP (a positive control) but does not affect cIP_3_‐evoked Ca^2+^ wave propagation. Previously, we showed that the broad spectrum gap junction blocker 18α‐glycyrrhetinic acid effectively blocked diffusion between endothelial cells but did not affect wave propagation [10]. Neither drug altered the cIP_3_‐evoked Ca^2+^ response within the photolysis region. Given that neither gap junctions nor diffusion appears to be involved, the question now arises: How are signals transmitted across the membranes from the initiating to the receiving cell?

The data presented suggests that IP_3_ is required to drive the propagating Ca^2+^ wave; therefore, IP_3_ must trigger signal propagation across the membrane, either directly or indirectly. Our results show that whilst IP_3_Rs are predominantly found throughout the cytoplasm, there is a peripheral, near‐membrane localization of IP_3_R clusters (Figure 6). This interpretation is consistent with previous studies reporting peripheral IP_3_R localization [53, 54, 55], as well as with the functional data presented in this manuscript. IP_3_R are now appreciated to support functions that are completely separate from the Ca^2+^ release activity of the channel. The functions documented thus far include triggering Ca^2+^ influx in staurosporine‐induced cell death [56], facilitating interactions of active STIM1 and Orai to promote store‐operated Ca^2+^ entry [53, 57] and providing structural roles in linking the endoplasmic reticulum and mitochondria [58]. Flux of Ca^2+^ through IP_3_R is not required to support each of these functions. Several other ionotropic receptors that previously have been thought to mediate their physiological effects *only* via the permeation of ions are now known to have important signaling features that do not arise from their ion flux capabilities. AMPA glutamate receptors and N‐methyl D‐aspartate receptors [59], the kainate receptor [60], the nicotinic acetylcholine receptor [61], the Kv1.3 potassium channel [62] and voltage‐dependent Ca^2+^ channels [63] all have signaling functions that are independent of ion flux through the channel pore. For example, activation of the ionotropic glutamate kainate receptor by the neurotransmitter gamma‐aminobutyric acid involves activation of PLC and protein kinase C via a Pertussis toxin–sensitive G protein rather than ion flux through the channel [60]. These studies highlight a diversity of signaling pathways that are activated and mediated by functionalities completely unrelated to the channels' ion carrying capabilities. It is therefore tempting to speculate that a receptor for IP_3_, which does not conduct ions, anchored at the interendothelial membrane, serves as the key link for propagating the signal to neighboring cells.

Rapid, accurate endothelial cell communication is fundamental to maintaining normal vascular function by transmitting information to dynamically regulate blood flow, mediate changes in vessel permeability, and to control immune responses. In the present study, the use of an *en face* blood vessels coupled with highly localized photolysis of caged IP_3_ and rapid, high‐resolution image acquisition permitted precise control of the time and location of IP_3_R activation within the intact endothelium. Each signal recorded outside the photolysis region arises as a direct consequence of the intercellular signaling cascade triggered by released IP_3_. Our results demonstrate a novel role for Gα_q/11_ subunit‐coupled GPCRs in mediating the propagation of Ca^2+^ waves within the endothelium. Importantly, our findings confirm that regenerative IP_3_ production, rather than Ca^2+^ itself, serves as the primary mechanism by which endothelial cells convey intercellular signals.

## Author Contributions

C.B., J.G.M.: conceptualization. C.B., C.W., M.D.L., X.Z., J.G.M.: methodology. C.B., J.G.M.: investigation. C.B., J.G.M.: writing – original draft. C.B., M.D.L., C.W., X.Z., J.G.M.: writing – review and editing. J.G.M., C.W., M.D.L., X.Z., C.B: funding acquisition.

## Conflicts of Interest

The authors declare no conflicts of interest.

## Supporting information


Figure S1.

Figure S2.

Video S1.

Video S2.

Video S3.

## Data Availability

All study data are included in the article and Supporting Information.
