# Supplementary figures and images for: Inside‐Out IP 3‐Mediated G Protein‐Coupled Receptor Activation Drives Intercellular Ca2+ Signaling in the Vascular Endothelium
Source: FASEB J. 2025 Jul 10;39(14):e70818. doi: 10.1096/fj.202500370RR (PMC12243451; doi:10.1096/fj.202500370RR)

# Supplementary Figure 1

*Localised High  $K^+$  puffing evoked depolarisation-induced contraction*

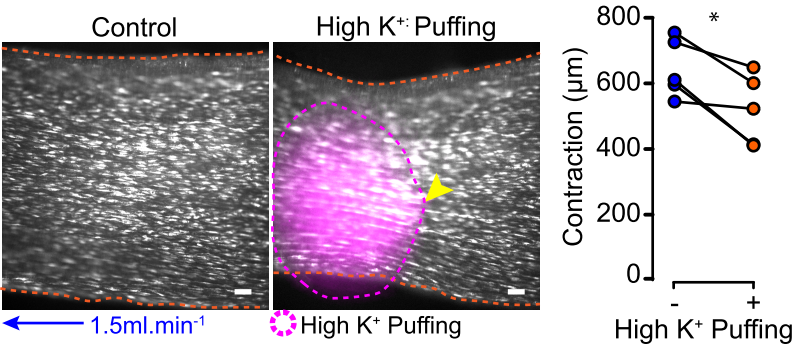

Supplement: Supplementary file 1 — Figure S1. [file FSB2-39-e70818-s003.pdf]
