# Supplementary material for: Inside‐Out IP 3‐Mediated G Protein‐Coupled Receptor Activation Drives Intercellular Ca2+ Signaling in the Vascular Endothelium
Source: FASEB J. 2025 Jul 10;39(14):e70818. doi: 10.1096/fj.202500370RR (PMC12243451; doi:10.1096/fj.202500370RR)

Supplementary Figure 2

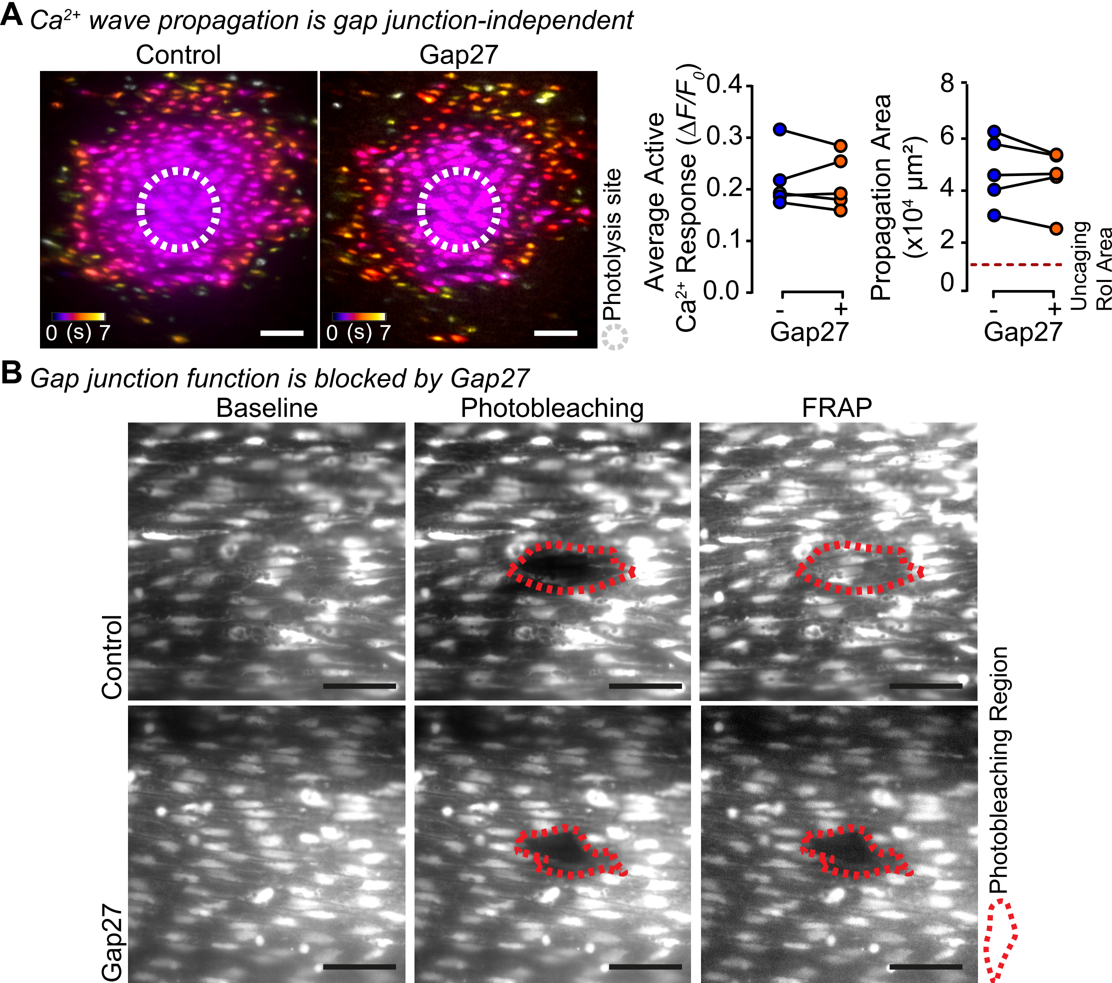

Supplement: Supplementary file 2 — Figure S2. [file FSB2-39-e70818-s001.pdf]
